# The pseudosymmetric structure of bis­(pentane-1,5-diaminium) iodide tris­(triiodide)

**DOI:** 10.1107/S1600536812014420

**Published:** 2012-04-06

**Authors:** Martin van Megen, Guido J. Reiss

**Affiliations:** a Institut für Anorganische Chemie und Strukturchemie, Lehrstuhl II: Material- und Strukturforschung, Heinrich-Heine-Universität Düsseldorf, Universitätsstrasse 1, D-40225 Düsseldorf, Germany

## Abstract

The asymmetric unit of the title compound, [H_3_N(CH_2_)_5_NH_3_]_2_I[I_3_]_3_ or 2C_5_H_16_N_2_^2+^·3I_3_^−^·I^−^, consists of two crystallographically independent pentane-1,5-diaminium dications and two triiodide anions in general positions besides two additional triiodide and two iodide anions located on twofold axes. The compound crystallizes in the centrosymmetric monoclinic space group *P*2/*n*. The structure refinement was handicapped by the pseudosymmetry (pseudo-centering) of the structure and by twinning. The crystal structure is composed of two alternate layers, which differ in their arrangement of the pentane-1,5-diaminium dications and the iodide/triiodide anions and which are connected *via* weak to medium–strong N—H⋯I hydrogen bonds, constructing a complex hydrogen-bonded network.

## Related literature

For general background to polyiodides, see: Svensson & Kloo (2003 ▶). For materials constructed by α,ω-diaminiumalkanes, see: Feng *et al.* (2000 ▶); Wiebcke (2002 ▶); Frank & Reiss (1997 ▶); Johnson *et al.* (2000 ▶). For applications of polyiodides, see: O’Regan & Grätzel (1991 ▶); Gorlov & Kloo (2008 ▶); Yang *et al.* (2011 ▶). For Raman spectroscopy of polyiodides, see: Deplano *et al.* (1999 ▶). For polyiodide-containing compounds with other stick-shaped cationic templates, see: Tebbe & Bittner (1995 ▶); Svensson *et al.* (2008 ▶); Abate *et al.* (2010 ▶); Meyer *et al.* (2010 ▶); Müller *et al.* (2010 ▶); García *et al.* (2011 ▶); Reiss & van Megen (2012 ▶). For polyiodide-containing α,ω-diaminiumalkanes compounds, see: Reiss & Engel (2002 ▶, 2004 ▶). For background to hydrogen bonds, see: Steiner (2002 ▶). For graph sets, see: Etter *et al.* (1990 ▶). For elemental analysis of iodine, see: Egli (1969 ▶). For programmes used to handle the pseudosymmetry, see: Sheldrick (2008 ▶); Spek (2009 ▶).
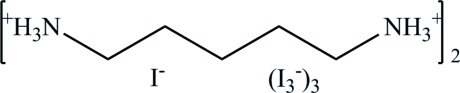


## Experimental

### 

#### Crystal data

2C_5_H_16_N_2_^2+^·3I_3_^−^·I^−^
*M* *_r_* = 1477.40
Monoclinic, 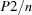

*a* = 11.24742 (18) Å
*b* = 24.4932 (3) Å
*c* = 11.49947 (16) Å
β = 99.5311 (14)°
*V* = 3124.21 (8) Å^3^
*Z* = 4
Mo *K*α radiation
μ = 9.92 mm^−1^
*T* = 110 K
0.35 × 0.13 × 0.03 mm

#### Data collection

Oxford Diffraction Xcalibur Eos diffractometer
Absorption correction: analytical [*CrysAlis PRO* (Oxford Diffraction, 2009 ▶), using a multi-faceted crystal model (Clark & Reid, 1995 ▶)] *T*_min_ = 0.184, *T*_max_ = 0.746
42753 measured reflections
5507 independent reflections
5106 reflections with *I* > 2σ(*I*)
*R*_int_ = 0.025

#### Refinement

*R*[*F* ^2^ > 2σ(*F* ^2^)] = 0.024
*wR*(*F* ^2^) = 0.048
*S* = 1.78
5507 reflections
260 parameters
12 restraints
H atoms treated by a mixture of independent and constrained refinement
Δρ_max_ = 0.89 e Å^−3^
Δρ_min_ = −0.85 e Å^−3^

### 

Data collection: *CrysAlis PRO* (Oxford Diffraction, 2009 ▶); cell refinement: *CrysAlis PRO*; data reduction: *CrysAlis PRO*; program(s) used to solve structure: *SHELXS97* (Sheldrick, 2008 ▶); program(s) used to refine structure: *SHELXL97* (Sheldrick, 2008 ▶); molecular graphics: *DIAMOND* (Brandenburg, 2011 ▶); software used to prepare material for publication: *publCIF* (Westrip, 2010 ▶).

## Supplementary Material

Crystal structure: contains datablock(s) I, global. DOI: 10.1107/S1600536812014420/br2194sup1.cif

Structure factors: contains datablock(s) I. DOI: 10.1107/S1600536812014420/br2194Isup2.hkl

Additional supplementary materials:  crystallographic information; 3D view; checkCIF report

## Figures and Tables

**Table 1:** Hydrogen-bond geometry (Å, °)

*D*—H⋯*A*	*D*—H	H⋯*A*	*D*⋯*A*	*D*—H⋯*A*
N1—H11⋯I2	0.89 (2)	2.87 (4)	3.632 (4)	144 (5)
N1—H12⋯I5^i^	0.89 (2)	3.00 (4)	3.757 (4)	145 (5)
N1—H13⋯I12^ii^	0.90 (2)	3.02 (5)	3.558 (4)	120 (4)
N2—H21⋯I5^ii^	0.90 (2)	3.02 (3)	3.786 (4)	145 (4)
N2—H22⋯I6	0.90 (2)	2.71 (3)	3.562 (4)	158 (4)
N3—H31⋯I6	0.89 (2)	2.85 (4)	3.607 (4)	144 (5)
N3—H32⋯I12	0.90 (2)	2.66 (3)	3.492 (4)	154 (5)
N4—H41⋯I9	0.90 (2)	2.82 (3)	3.634 (4)	153 (4)
N4—H42⋯I8	0.90 (2)	2.70 (2)	3.564 (4)	162 (4)
N4—H43⋯I11^iii^	0.90 (2)	2.94 (4)	3.621 (4)	134 (4)

Symmetry codes: (i) 
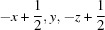
; (ii) 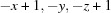; (iii) 
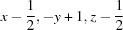
.

**Fig. 1.**
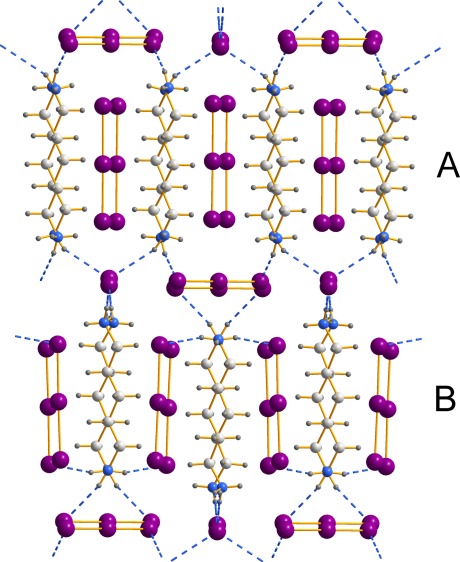
: View along [001] on the structure of the title compound. Showing the hydrogen-bonded motifs arranged as two alternate layers (atom radii are drawn at arbitrary size; only classical hydrogen bonds are shown).

**Fig. 2.**
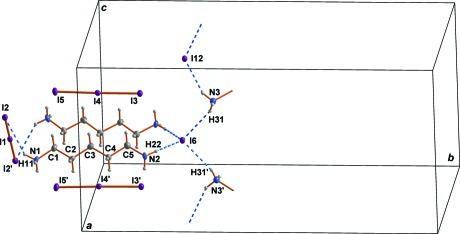
: Showing the basic structural motif of the A layer (symmetry code: ' = 0.5 - x, y, 0.5 - z, displacement ellipsoids are drawn at the 70% probability level; hydrogen atoms are drawn as spheres with arbitrary radii; only classical hydrogen bonds are shown).

**Fig. 3.**
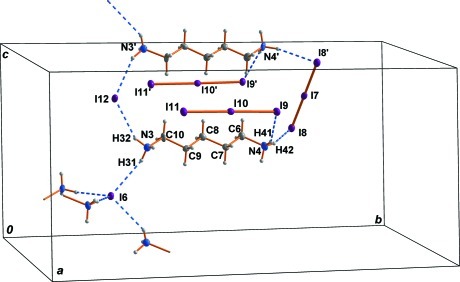
: Showing the basic structural motif of the B layer (symmetry code: ' = 0.5 - x, y, 1.5 - z, displacement ellipsoids are drawn with 70% probability; hydrogen atoms are drawn as spheres with arbitrary radii; only classical hydrogen bonds are shown).

**Fig. 4.**
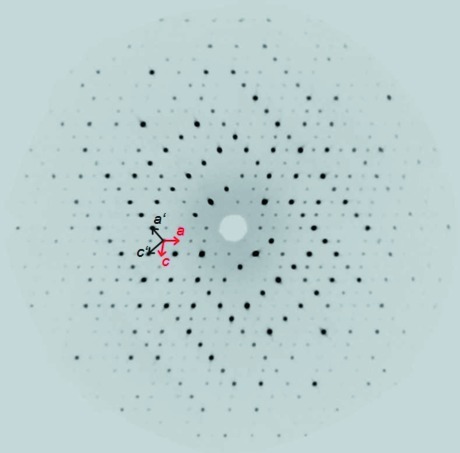
: Reconstruction (Oxford Diffraction, 2009) of the h-2 l layer of the reciprocal lattice of the title compound; a, c: true cell (red); a', c': pseudosymmetric cell (black).

**Fig. 5.**
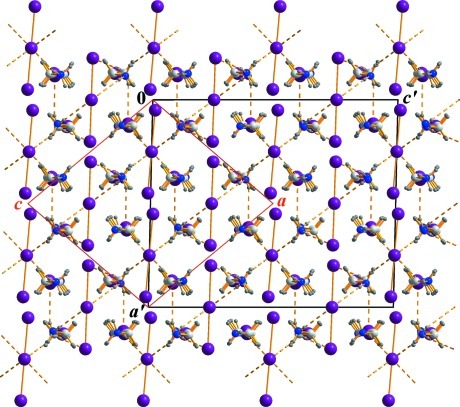
: View along [010] on the pseudosymmetric title structure; a, c: true cell (red); a', c': pseudosymmetric cell (black).

## supplementary crystallographic information

### Comment

There is a general interest in diaminiumalkane iodides and polyiodides as they are well known for having a significant influence on the redox chemistry in dye-sensitized solar cells (O'Regan & Grätzel, 1991; Gorlov & Kloo, 2008; Yang *et al.*, 2011). The semi-flexible, stick-shaped α,ω-diaminiumalkane dications have proved to be potent templates for *crystal engineering* with a wide field of application, *e. g.* for the synthesis of layered structures of aluminium and zinc phosphates (Feng *et al.*, 2000; Wiebcke, 2002), for the encapsulation of hydronium cations with unusual topology in hydrogen-bonded frameworks (Frank & Reiss, 1997) and for connecting metal clusters as special spacers (Johnson *et al.*, 2000). In the recent past several groups have also synthesized new polyiodides using stick-shaped cationic templates whose lengths and shapes fit with the structures of the polyiodides (Tebbe & Bittner, 1995; Abate *et al.*, 2010; Meyer *et al.*, 2010; García *et al.*, 2011; Reiss & van Megen, 2012). This selective and robust synthetic protocol for solid polyiodides is now termed *dimensional caging* (Svensson *et al.*, 2008). Especially the α,ω-diaminiumalkane dications have successfully been used for the *dimensional caging* of polyiodides (Reiss & Engel, 2002; Reiss & Engel, 2004). This contribution presents the crystal structure of a salt composed of pentane-1,5-diaminium dications and iodide and triiodide anions.

The asymmetric unit of the title compound consists of two crystallographically independent pentane-1,5-diaminium dications and two triiodides in general positions. In addition to that there are two more triiodides and two iodide anions all located on twofold axes. The organic dications exhibit an *all-trans* conformation within the experimental uncertainties. The crystallographically independent dications are found in two different, alternate layers which are connected by weak to medium strong N–H···I hydrogen-bonds (Steiner, 2002). The pentane-1,5-diaminium dications and the different types of anions construct a complex hydrogen-bonded framework. Generally the N–H···I hydrogen bonds accepted by the iodide anions are, as expected, shorter than those accepted by the triiodide anions. However, there is also one triiodide anion which is not involved in any classical hydrogen bonding, but it is integrated in the structure by weak H···I contacts (Fig. 1).

The basic hydrogen-bonded structural motif in both layers consists of two cations, one iodide and one triiodide arranged as a ring (Fig. 2 and Fig. 3). In these hydrogen-bonded rings the iodide anion accepts two hydrogen bonds and the triiodide anion accepts one hydrogen bond at each terminal iodine atom (graph set: *R*^3^_4_(22); Etter, 1990). In the A layer the iodide anion accepts two more hydrogen bonds of neighbouring aminium groups whereas the triiodide anion is not further connected (Fig. 2, Table 1). In contrast to that in layer B the iodide and the triiodide anion of the basic hydrogen-bonded ring motif are not involved in further hydrogen bonding. The connection to neighbouring units in this case is performed by the aminium groups (Fig. 3, Table 1). In both layers triiodide anions (I3–I4–I5; I9–I10–I11) are arranged parallel to the rod-shaped cations. The inclusion of these triiodides can be understood as a typical encapsulation of a small polyiodide (Abate *et al.*, 2010; Müller *et al.*, 2010; García *et al.*, 2011).

All triiodide anions in this compound are nearly linear and symmetric with bond lengths and angles in the expected ranges (Svensson & Kloo, 2003). Furthermore the Raman spectroscopic results are in excellent agreement with those of the crystal structure analysis. For a centrosymmetric triiodide anion with *D*_∞_*_h_* symmetry one Raman active band from the centrosymmetric stretching vibration is predicted at ~110 cm^-1^ by selection rules (Deplano *et al.*, 1999). The experimental Raman spectrum of the title compound shows one very strong band at 110 cm^-1^.

The whole structure determination is affected by pseudosymmetry problems. The diffraction pattern shows weak superstructure reflections besides the main reflections (Fig. 4). The ADDSYM option of the *PLATON* programme (Spek, 2009) detects a centering of most non-hydrogen atoms which produces a B-Alert using the IUCR-CheckCif programme. A view along [010] shows the title structure (Fig. 5) with the true monoclinic cell (red) and the pseudo-orthorhombic cell (black). From all the non-hydrogen atom positions in the asymmetric unit, only two iodide anion positions do not fit with a face-centered description of the structure. In the projection along [010] the deviation from the higher symmetric description is marginal. Fig. 4 and Fig. 5 document the difficulties which arose during the data collection and the structure refinement. As the final structural model does not reveal any disorder, including the hydrogen atoms, a description in a higher symmetric model accepting a disorder has definitively been ruled out.

### Experimental

The title compound, [H_3_N(CH_2_)_5_NH_3_]_2_I[I_3_]_3_, was prepared by dissolving 0.16 g (1.6 mmol) 1,5-diaminopentane and 0.81 g (3.2 mmol) iodine in 10 ml concentrated (57%) hydroiodic acid. Heating to 373 K yielded a dark coloured solution. Upon slow cooling to room temperature, dark-red, shiny crystals were formed at the bottom of the reaction vessel within one to two days.

The Raman spectrum was measured using a Bruker *MULTIRAM* spectrometer (Nd:YAG-laser at 1064 nm; InGaAs-detector); 300–70 cm^-1^: 216(w), 110(*vs*). – IR spectroscopic data were collected on a *Digilab FT3500* spectrometer using a MIRacle ATR unit (Pike Technologies); 4000–560 cm^-1^: 3358(*vs*, br), 3197(*vs*, sh) 3161(*vs*), 2980(*s*), 2953(*s*), 2901(*s*), 2851(*s*), 2430(w), 2354(w), 1615(m, br), 1558(*m*), 1455(*m*), 1439(*m*), 1155(w), 948(w), 796(w), 721(w). – Elemental analyses (C, H, N) were performed with a HEKA-Tech *Euro EA3000* instrument; C_10_H_32_N_4_I_10_ (1477.44): calcd. C 8.13, H 2.18, N 3.79; found C 7.81, H 2.02, N 3.77. – Elemental analysis of iodine: In a typical experiment 100 mg of the title compound were dissolved in 15 ml of a water/acetone (10:1) mixture. After adding some drops of acetic acid and heating up to approximately 373 K zinc powder was added until the solution turns colourless. Filtering off the surplus of zinc yielded a clear solution which was analyzed by a classical precipitation titration (AgNO_3_ solution (0.1 mol/L); potentiometric endpoint; Ag/AgCl//Ag electrodes) (Egli, 1969): calcd. 85.9%; found 84.0%.

### Refinement

A crystal of the title compound was mounted in the cold stream of an Oxford four-circle diffractometer. Most crystals were seriously twinned. The irradiation time was raised to outgrow the weak superstructure reflections from the background. A closer examination showed that there was also a small amount of a twin component attached to the single-crystal (below 5% of reflections of the peak hunting table). The true monoclinic cell could be transformed to an approximately orthorombic cell (14.69 Å, 24.49 Å, 17.36 Å, 90.0 °, 91.3°, 90.0 °) which has been ruled out for the angle deviation and the high *R*_int_ (>0.24) value. The secondary structure solution and the refinement were complicated due to the pseudosymmetry effects. Fig. 4 shows a reconstruction from the data collection images of the h-2 l layer of the reciprocal lattice. This figure shows abundantly clear the pseudosymmetry expressed as very strong reflections belonging to a higher translational symmetry (basis structure) and weak superstructure reflections defining the true structure. The refinement of the anisotropic displacement parameters for the nitrogen and carbon atoms only succeeded with the parameters kept roughly isotropical (ISOR option of the *SHELX* programme; Sheldrick, 2008). The hydrogen atoms of the CH_2_ groups were included using a riding model. The *U*_iso_(H) values were set 1.2 times of their parent atoms. Refinement of this structural model yielded all 12 missing hydrogen atom positions of the aminium groups. In the latest stages of refinement the hydrogen atom positions of these were refined with their N—H distances softly restrained with a common U value for each group. In the final refinements it was possible to omit the restraints on the anisotropic displacement parameters. For the most disagreeable reflections in the Fo/Fc statistic it was observed that the Fo value is always too large. This finding must be attributed to the fact that a small twin component added its intensity to some reflections.

### Crystal data

**Table:**

2C_5_H_16_N_2_^2+^·3I_3_^−^·I^−^	*F*(000) = 2600
*M**_r_* = 1477.40	*D*_x_ = 3.141 Mg m^−^^3^
Monoclinic, *P*2/*n*	Mo *K*α radiation, λ = 0.71073 Å
Hall symbol: -P 2yac	Cell parameters from 34080 reflections
*a* = 11.24742 (18) Å	θ = 2.9–32.9°
*b* = 24.4932 (3) Å	µ = 9.92 mm^−^^1^
*c* = 11.49947 (16) Å	*T* = 110 K
β = 99.5311 (14)°	Plate, dark-red
*V* = 3124.21 (8) Å^3^	0.35 × 0.13 × 0.03 mm
*Z* = 4	

### Data collection

**Table:**

Oxford Diffraction Xcalibur Eos diffractometer	5507 independent reflections
Radiation source: fine-focus sealed tube	5106 reflections with *I* > 2σ(*I*)
Graphite monochromator	*R*_int_ = 0.025
Detector resolution: 16.2711 pixels mm^-1^	θ_max_ = 25.0°, θ_min_ = 2.9°
ω scan	*h* = −13→13
Absorption correction: analytical [*CrysAlis PRO* (Oxford Diffraction, 2009), using a multi-faceted crystal model (Clark & Reid, 1995)]	*k* = −29→29
*T*_min_ = 0.184, *T*_max_ = 0.746	*l* = −13→13
42753 measured reflections	

### Refinement

**Table:**

Refinement on *F*^2^	Secondary atom site location: difference Fourier map
Least-squares matrix: full	Hydrogen site location: inferred from neighbouring sites
*R*[*F*^2^ > 2σ(*F*^2^)] = 0.024	H atoms treated by a mixture of independent and constrained refinement
*wR*(*F*^2^) = 0.048	*w* = 1/[σ^2^(*F*_o_^2^) + (0.010*P*)^2^ + 2.*P*] where *P* = (*F*_o_^2^ + 2*F*_c_^2^)/3
*S* = 1.78	(Δ/σ)_max_ = 0.002
5507 reflections	Δρ_max_ = 0.89 e Å^−^^3^
260 parameters	Δρ_min_ = −0.85 e Å^−^^3^
12 restraints	Extinction correction: *SHELXL97* (Sheldrick, 2008), Fc^*^=kFc[1+0.001xFc^2^λ^3^/sin(2θ)]^-1/4^
Primary atom site location: structure-invariant direct methods	Extinction coefficient: 0.000079 (7)

### Special details

**Table:**

Experimental. Absorption correction: CrysAlis PRO, Oxford Diffraction Ltd., Version 1.171.34.44. Analytical numeric absorption correction using a multifaceted crystal model (Clark & Reid, 1995).
Geometry. All e.s.d.'s (except the e.s.d. in the dihedral angle between two l.s. planes) are estimated using the full covariance matrix. The cell e.s.d.'s are taken into account individually in the estimation of e.s.d.'s in distances, angles and torsion angles; correlations between e.s.d.'s in cell parameters are only used when they are defined by crystal symmetry. An approximate (isotropic) treatment of cell e.s.d.'s is used for estimating e.s.d.'s involving l.s. planes.
Refinement. Refinement of *F*^2^ against ALL reflections. The weighted *R*-factor *wR* and goodness of fit *S* are based on *F*^2^, conventional *R*-factors *R* are based on *F*, with *F* set to zero for negative *F*^2^. The threshold expression of *F*^2^ > σ(*F*^2^) is used only for calculating *R*-factors(gt) *etc*. and is not relevant to the choice of reflections for refinement. *R*-factors based on *F*^2^ are statistically about twice as large as those based on *F*, and *R*- factors based on ALL data will be even larger.

### Fractional atomic coordinates and isotropic or equivalent isotropic displacement parameters (Å^2^)

**Table:**

	*x*	*y*	*z*	*U*_iso_*/*U*_eq_	
I1	0.2500	−0.244327 (17)	0.2500	0.01394 (10)	
I2	0.43884 (3)	−0.243981 (13)	0.46114 (2)	0.01481 (8)	
I3	0.21847 (3)	0.122482 (12)	0.52226 (3)	0.01339 (8)	
I4	0.21879 (3)	0.003697 (12)	0.52343 (2)	0.01055 (8)	
I5	0.21699 (3)	−0.116102 (12)	0.52918 (3)	0.01481 (8)	
I6	0.2500	0.239763 (18)	0.2500	0.01396 (10)	
I7	0.2500	0.742057 (18)	0.7500	0.01391 (10)	
I8	0.05106 (3)	0.741845 (13)	0.54698 (3)	0.01517 (8)	
I9	0.51718 (3)	0.623905 (13)	0.71356 (3)	0.01481 (8)	
I10	0.52267 (3)	0.505510 (12)	0.71916 (2)	0.01173 (8)	
I11	0.53337 (3)	0.385420 (13)	0.72274 (3)	0.01511 (8)	
I12	0.2500	0.253336 (18)	0.7500	0.01617 (10)	
N1	0.5149 (4)	−0.15499 (17)	0.2316 (4)	0.0158 (9)	
H11	0.496 (5)	−0.1876 (13)	0.259 (5)	0.037 (10)*	
H12	0.487 (5)	−0.154 (2)	0.155 (2)	0.037 (10)*	
H13	0.5955 (19)	−0.152 (2)	0.249 (5)	0.037 (10)*	
N2	0.5001 (4)	0.15009 (17)	0.2556 (4)	0.0147 (9)	
H21	0.574 (3)	0.158 (2)	0.295 (4)	0.024 (8)*	
H22	0.453 (4)	0.1790 (15)	0.266 (4)	0.024 (8)*	
H23	0.496 (5)	0.148 (2)	0.1766 (18)	0.024 (8)*	
N3	0.2715 (4)	0.33075 (17)	0.4993 (4)	0.0160 (9)	
H31	0.239 (5)	0.320 (2)	0.427 (3)	0.037 (10)*	
H32	0.262 (5)	0.3018 (16)	0.545 (4)	0.037 (10)*	
H33	0.3522 (19)	0.334 (2)	0.513 (5)	0.037 (10)*	
N4	0.2500 (4)	0.63571 (17)	0.4891 (4)	0.0142 (9)	
H41	0.326 (2)	0.640 (2)	0.525 (4)	0.024 (8)*	
H42	0.211 (4)	0.6674 (13)	0.496 (4)	0.024 (8)*	
H43	0.242 (5)	0.629 (2)	0.4116 (19)	0.024 (8)*	
C1	0.4644 (4)	−0.10655 (19)	0.2854 (4)	0.0153 (10)	
H1A	0.4858	−0.1084	0.3706	0.018*	
H1B	0.3771	−0.1069	0.2655	0.018*	
C2	0.5124 (4)	−0.05421 (19)	0.2416 (4)	0.0126 (10)	
H2A	0.4923	−0.0528	0.1563	0.015*	
H2B	0.5996	−0.0537	0.2627	0.015*	
C3	0.4602 (4)	−0.00432 (18)	0.2939 (4)	0.0137 (10)	
H3A	0.3731	−0.0049	0.2726	0.016*	
H3B	0.4800	−0.0058	0.3792	0.016*	
C4	0.5083 (4)	0.04858 (19)	0.2507 (4)	0.0112 (10)	
H4A	0.5950	0.0501	0.2756	0.013*	
H4B	0.4922	0.0493	0.1652	0.013*	
C5	0.4506 (4)	0.09804 (19)	0.2983 (4)	0.0156 (10)	
H5A	0.3641	0.0968	0.2727	0.019*	
H5B	0.4661	0.0972	0.3838	0.019*	
C6	0.1961 (4)	0.58547 (19)	0.5357 (4)	0.0163 (10)	
H6A	0.1093	0.5856	0.5111	0.020*	
H6B	0.2128	0.5854	0.6212	0.020*	
C7	0.2497 (4)	0.53499 (19)	0.4888 (4)	0.0128 (10)	
H7A	0.3367	0.5360	0.5113	0.015*	
H7B	0.2312	0.5350	0.4034	0.015*	
C8	0.2013 (4)	0.4825 (2)	0.5357 (4)	0.0146 (10)	
H8A	0.2204	0.4822	0.6211	0.018*	
H8B	0.1143	0.4815	0.5136	0.018*	
C9	0.2558 (4)	0.43232 (19)	0.4869 (4)	0.0125 (10)	
H9A	0.2336	0.4317	0.4017	0.015*	
H9B	0.3430	0.4341	0.5059	0.015*	
C10	0.2115 (4)	0.38037 (18)	0.5385 (4)	0.0162 (11)	
H10A	0.2277	0.3825	0.6239	0.019*	
H10B	0.1249	0.3773	0.5141	0.019*	

### Atomic displacement parameters (Å^2^)

**Table:**

	*U*^11^	*U*^22^	*U*^33^	*U*^12^	*U*^13^	*U*^23^
I1	0.0179 (2)	0.0086 (2)	0.0172 (2)	0.000	0.00819 (18)	0.000
I2	0.01940 (17)	0.01083 (17)	0.01497 (15)	0.00174 (12)	0.00510 (13)	0.00131 (12)
I3	0.01559 (16)	0.00995 (16)	0.01443 (15)	0.00031 (12)	0.00189 (13)	0.00010 (12)
I4	0.01090 (16)	0.01066 (16)	0.00983 (15)	0.00076 (11)	0.00097 (13)	−0.00075 (11)
I5	0.01700 (16)	0.00901 (16)	0.01688 (16)	0.00144 (12)	−0.00170 (13)	−0.00120 (12)
I6	0.0174 (2)	0.0088 (2)	0.0169 (2)	0.000	0.00622 (18)	0.000
I7	0.0179 (2)	0.0094 (2)	0.0164 (2)	0.000	0.00837 (18)	0.000
I8	0.01680 (16)	0.01138 (17)	0.01794 (16)	0.00001 (12)	0.00468 (13)	0.00029 (12)
I9	0.01788 (17)	0.01071 (17)	0.01540 (15)	0.00194 (12)	0.00146 (13)	−0.00085 (12)
I10	0.01125 (16)	0.01285 (17)	0.01060 (15)	0.00072 (11)	0.00034 (13)	−0.00022 (12)
I11	0.01647 (16)	0.01090 (17)	0.01654 (16)	0.00027 (12)	−0.00146 (13)	−0.00011 (12)
I12	0.0243 (2)	0.0087 (2)	0.0173 (2)	0.000	0.00892 (18)	0.000
N1	0.018 (2)	0.008 (2)	0.021 (2)	−0.0010 (18)	0.0036 (19)	0.0046 (18)
N2	0.019 (2)	0.007 (2)	0.017 (2)	0.0005 (17)	0.0021 (19)	−0.0019 (18)
N3	0.020 (2)	0.012 (2)	0.016 (2)	0.0002 (18)	0.0045 (19)	0.0004 (17)
N4	0.014 (2)	0.009 (2)	0.019 (2)	0.0044 (17)	0.0027 (18)	−0.0021 (18)
C1	0.020 (3)	0.010 (3)	0.017 (2)	−0.001 (2)	0.005 (2)	0.002 (2)
C2	0.012 (2)	0.013 (3)	0.012 (2)	−0.0026 (19)	0.002 (2)	0.0027 (19)
C3	0.014 (2)	0.014 (3)	0.013 (2)	−0.0008 (19)	0.004 (2)	0.0016 (19)
C4	0.011 (2)	0.010 (2)	0.012 (2)	0.0002 (19)	0.0018 (19)	−0.0010 (19)
C5	0.016 (2)	0.013 (3)	0.018 (2)	0.000 (2)	0.004 (2)	−0.001 (2)
C6	0.019 (3)	0.012 (3)	0.019 (2)	−0.002 (2)	0.005 (2)	−0.002 (2)
C7	0.014 (2)	0.012 (3)	0.012 (2)	−0.0004 (19)	0.000 (2)	0.0002 (19)
C8	0.016 (2)	0.013 (3)	0.015 (2)	0.000 (2)	0.003 (2)	−0.002 (2)
C9	0.013 (2)	0.014 (3)	0.010 (2)	−0.0008 (19)	0.0016 (19)	0.0027 (19)
C10	0.023 (3)	0.005 (2)	0.021 (2)	0.000 (2)	0.005 (2)	−0.002 (2)

### Geometric parameters (Å, º)

**Table:**

I1—I2	2.9494 (3)	C1—H1B	0.9700
I1—I2^i^	2.9494 (3)	C2—C3	1.522 (6)
I3—I4	2.9095 (4)	C2—H2A	0.9700
I4—I5	2.9352 (4)	C2—H2B	0.9700
I7—I8^ii^	2.9542 (3)	C3—C4	1.519 (6)
I7—I8	2.9542 (3)	C3—H3A	0.9700
I9—I10	2.9010 (4)	C3—H3B	0.9700
I10—I11	2.9439 (4)	C4—C5	1.519 (6)
N1—C1	1.493 (6)	C4—H4A	0.9700
N1—H11	0.892 (19)	C4—H4B	0.9700
N1—H12	0.888 (19)	C5—H5A	0.9700
N1—H13	0.899 (19)	C5—H5B	0.9700
N2—C5	1.506 (6)	C6—C7	1.513 (7)
N2—H21	0.898 (19)	C6—H6A	0.9700
N2—H22	0.904 (19)	C6—H6B	0.9700
N2—H23	0.903 (19)	C7—C8	1.528 (7)
N3—C10	1.496 (6)	C7—H7A	0.9700
N3—H31	0.891 (19)	C7—H7B	0.9700
N3—H32	0.904 (19)	C8—C9	1.522 (7)
N3—H33	0.899 (19)	C8—H8A	0.9700
N4—C6	1.509 (6)	C8—H8B	0.9700
N4—H41	0.895 (19)	C9—C10	1.522 (6)
N4—H42	0.900 (19)	C9—H9A	0.9700
N4—H43	0.898 (19)	C9—H9B	0.9700
C1—C2	1.510 (6)	C10—H10A	0.9700
C1—H1A	0.9700	C10—H10B	0.9700
			
I2—I1—I2^i^	179.67 (2)	C2—C3—H3B	109.2
I3—I4—I5	178.805 (15)	H3A—C3—H3B	107.9
I8^ii^—I7—I8	179.80 (2)	C5—C4—C3	111.4 (4)
I9—I10—I11	178.713 (15)	C5—C4—H4A	109.3
C1—N1—H11	116 (4)	C3—C4—H4A	109.3
C1—N1—H12	107 (4)	C5—C4—H4B	109.3
H11—N1—H12	108 (5)	C3—C4—H4B	109.3
C1—N1—H13	107 (4)	H4A—C4—H4B	108.0
H11—N1—H13	106 (5)	N2—C5—C4	110.8 (4)
H12—N1—H13	114 (5)	N2—C5—H5A	109.5
C5—N2—H21	112 (3)	C4—C5—H5A	109.5
C5—N2—H22	111 (3)	N2—C5—H5B	109.5
H21—N2—H22	106 (5)	C4—C5—H5B	109.5
C5—N2—H23	109 (3)	H5A—C5—H5B	108.1
H21—N2—H23	114 (5)	N4—C6—C7	109.5 (4)
H22—N2—H23	104 (5)	N4—C6—H6A	109.8
C10—N3—H31	113 (4)	C7—C6—H6A	109.8
C10—N3—H32	111 (4)	N4—C6—H6B	109.8
H31—N3—H32	104 (5)	C7—C6—H6B	109.8
C10—N3—H33	112 (4)	H6A—C6—H6B	108.2
H31—N3—H33	116 (5)	C6—C7—C8	112.1 (4)
H32—N3—H33	101 (5)	C6—C7—H7A	109.2
C6—N4—H41	110 (3)	C8—C7—H7A	109.2
C6—N4—H42	116 (3)	C6—C7—H7B	109.2
H41—N4—H42	108 (5)	C8—C7—H7B	109.2
C6—N4—H43	103 (3)	H7A—C7—H7B	107.9
H41—N4—H43	114 (5)	C9—C8—C7	111.2 (4)
H42—N4—H43	106 (5)	C9—C8—H8A	109.4
N1—C1—C2	110.8 (4)	C7—C8—H8A	109.4
N1—C1—H1A	109.5	C9—C8—H8B	109.4
C2—C1—H1A	109.5	C7—C8—H8B	109.4
N1—C1—H1B	109.5	H8A—C8—H8B	108.0
C2—C1—H1B	109.5	C10—C9—C8	110.7 (4)
H1A—C1—H1B	108.1	C10—C9—H9A	109.5
C1—C2—C3	111.6 (4)	C8—C9—H9A	109.5
C1—C2—H2A	109.3	C10—C9—H9B	109.5
C3—C2—H2A	109.3	C8—C9—H9B	109.5
C1—C2—H2B	109.3	H9A—C9—H9B	108.1
C3—C2—H2B	109.3	N3—C10—C9	111.6 (4)
H2A—C2—H2B	108.0	N3—C10—H10A	109.3
C4—C3—C2	112.0 (4)	C9—C10—H10A	109.3
C4—C3—H3A	109.2	N3—C10—H10B	109.3
C2—C3—H3A	109.2	C9—C10—H10B	109.3
C4—C3—H3B	109.2	H10A—C10—H10B	108.0
			
N1—C1—C2—C3	179.0 (4)	N4—C6—C7—C8	178.4 (4)
C1—C2—C3—C4	179.8 (4)	C6—C7—C8—C9	179.6 (4)
C2—C3—C4—C5	177.0 (4)	C7—C8—C9—C10	177.4 (4)
C3—C4—C5—N2	179.5 (4)	C8—C9—C10—N3	−175.4 (4)

Symmetry codes: (i) −*x*+1/2, *y*, −*z*+1/2; (ii) −*x*+1/2, *y*, −*z*+3/2.

### Hydrogen-bond geometry (Å, º)

**Table:**

*D*—H···*A*	*D*—H	H···*A*	*D*···*A*	*D*—H···*A*
N1—H11···I2	0.89 (2)	2.87 (4)	3.632 (4)	144 (5)
N1—H12···I5^i^	0.89 (2)	3.00 (4)	3.757 (4)	145 (5)
N1—H13···I12^iii^	0.90 (2)	3.02 (5)	3.558 (4)	120 (4)
N2—H21···I5^iii^	0.90 (2)	3.02 (3)	3.786 (4)	145 (4)
N2—H22···I6	0.90 (2)	2.71 (3)	3.562 (4)	158 (4)
N3—H31···I6	0.89 (2)	2.85 (4)	3.607 (4)	144 (5)
N3—H32···I12	0.90 (2)	2.66 (3)	3.492 (4)	154 (5)
N4—H41···I9	0.90 (2)	2.82 (3)	3.634 (4)	153 (4)
N4—H42···I8	0.90 (2)	2.70 (2)	3.564 (4)	162 (4)
N4—H43···I11^iv^	0.90 (2)	2.94 (4)	3.621 (4)	134 (4)

Symmetry codes: (i) −*x*+1/2, *y*, −*z*+1/2; (iii) −*x*+1, −*y*, −*z*+1; (iv) *x*−1/2, −*y*+1, *z*−1/2.
